# Desmosomes in squamous cell carcinomas

**DOI:** 10.48101/ujms.v127.8250

**Published:** 2022-01-25

**Authors:** Vitorino Modesto dos Santos, Lister Arruda Modesto dos Santos, Taciana Arruda Modesto Sugai

**Affiliations:** Department of Medicine, Armed Forces Hospital and Catholic University of Brasília-DF, Brazil; General Surgery Department, State Worker’s Hospital, São Paulo-SP, Brazil; American Society of Neurophysiology, and Dermatologist of Brasília-DF, Brazil

Dear Editor

The differential diagnosis of adenocarcinomas and squamous cell cancer may present a challenging task in diagnostic pathology. We appreciated the informative study by Galindo et al. recently published in the *Upsala Journal of Medical Science* evaluating the expression of three desmosomal proteins in non-small cell lung cancer (1). Desmosomes are structures of the plasma membrane participating in intercellular adhesion; and proteins of the plakophilin (PKP) family, like desmoplakin and plakoglobin, play a functional role in the desmosome plaques, with PKP1 as a major component (1). Another desmosomal protein, desmoglein 3 (DSG3), can act as tumor suppressor and is downregulated in stages of invasion and metastasis. In addition, the study included several established markers for NSCLC differentiation (CK5/6, p40, p63, CK7, TTF1, and Napsin A) as well as the novel marker keratin 15 (KRT15) (1). Protein staining related to the desmosome plaque has been utilized to establish the diagnosis of squamous cell carcinoma (SCC), as the example of pulmonary and head and neck regions. Gene sequences corresponding to the desmosome plaque-related proteins PKP1, DSG3, and KRT15 are differentially expressed in primary pulmonary adenocarcinoma and SCC (1). The established and novel markers were evaluated to distinguish pulmonary SCCs (*n* = 41) from adenocarcinomas (*n* = 44) in small lung biopsies. Their respective specificities for correctly diagnosed SCC were 97.4%, 94.6%, and 100% (g). Using CK5/6, p63, and PKP1 correctly determined SCC in 97.6% of cases. Moreover, PKP1 and DSG3 expression showed association with the patient outcome (1).

In addition to desmosome proteins, other tools have been used for diagnosis, clinical management, and prognostication of either suspicious or confirmed cases of SCC (1–5). PERP (apoptosis effector of p53 related to PMP22) can stabilize desmosomes and suppress the SCC development, and the local control of SCC becomes inefficient if it is absent (2). Holmes et al. analyzed the 2-year cumulative incidence of local recurrence in 44 patients with SCC and described 44.4% recurrence for the PERP-negative and 16.4% for the PERP-positive group. The authors highlighted that PERP loss at SCC surgical margins is a predictor of relapses (2). Overexpression of kallikrein 7 (KLK7) in oral SCC can promote degradation of desmosomes, favoring local tumor invasion and metastases to the lymph nodes (3). Kumar et al. studied KLK7 expression in 30 patients with oral SCCs compared to normal controls. KLK7 was differentially expressed in all tumors varying with the histopathological grades and clinical stages. KLK7 expression increased from SCC low to high grade and from clinical stages 1–4, and this enhanced activity promoted over-desquamation and was related to poor prognosis (3). The authors emphasized the potential role of KLK7 as a useful diagnostic marker for oral SCC to monitor response to therapy, or as a candidate target for the treatment of refractory cases (3).

Also electron microscopy was applied to further analyze the role of intercellular desmosomes in tissue samples with the aim to evaluate invasive and proliferative capacity of SCC (4, 5). Murakami et al. studied the morphology of three-dimensional (3D) cultured cells using a tissueoid cell culture system (Cell Bed scaffold) using silicate fibers as scaffolds providing structures similar to that occurring in cancer tissues (4). The scaffold acts as an extracellular matrix substrate for cell growth and the in vivo 3D structures. SCC cells were coated with collagen I, III, and IV to evaluate the role of extracellular matrix proteins. Coating with collagen IV mimicked cell–matrix interactions of the in vivo microenvironment; so, they found that SCCs invasiveness and proliferation enhance with the presence of collagen (4). Well-differentiated tumor cells cultured in this 3D system showed their typical characteristics, the luminal formation in adenocarcinomas and stratification and keratinization in SCCs (4).

In this context, we like to highlight the ultrastructural research in samples of SCCs from tongue (35.7%), oral floor (35.7%), gingiva (21.4%), and lip (7.1%) done in our group (5). We studied the relationship of tumor histological grading with the desmosome size. The lengths of 1,507 desmosome profiles were evaluated in oral SCC of high (21.4%), moderate (50%), and poor (28.6%) differentiation. The desmosomes were grouped as normal (≤ 0.5 μm), large (0.5–1.0 μm), and giant (≥ 1.0 μm). The number of desmosomes by the number of histological grading of the SCCs was as follows: 465/3 well-differentiated, 673/7 moderately differentiated; and 369/4 poorly differentiated tumors. The quantitative distribution by desmosome size was as follows: 114 normal, 251 large, and 100 giant in well-differentiated SCCs; 266 normal, 316 large, and 91 giant in moderately differentiated SCCs; and 125 normal, 160 large, and 84 giant in poorly differentiated SCCs. The median size (μm) of desmosomes according to the histological grading of the SCCs was as follows: 0.70 (well-differentiated), 0.56 (moderately differentiated), and 0.67 (poorly differentiated). There was homogeneity in desmosome sizes in the histological grading groups of SCCs: well-differentiated (100%), moderately differentiated (95.2%), and poorly differentiated (50%). Worthy of note was the correlation between the length of desmosomes and the SCC histological grading, which merits further research to disclose some prognostic role related to the pointed homogeneity (5).

The aim of the comments addressed herein is to emphasize the role of desmosomes in the tumor biology of SCC and to stimulate further studies to optimize the diagnostic and management of SCCs.


Vitorino Modesto dos Santos Department of Medicine, Armed Forces Hospital and Catholic University of Brasília-DF, Brazil
Lister Arruda Modesto dos Santos General Surgery Department, State Worker’s Hospital, São Paulo-SP, Brazil
Taciana Arruda Modesto Sugai American Society of Neurophysiology, and Dermatologist of Brasília-DF, Brazil

